# Stress inoculation in mice induces global resilience

**DOI:** 10.1038/s41398-020-00889-0

**Published:** 2020-06-19

**Authors:** Sarah Ayash, Ulrich Schmitt, David M. Lyons, Marianne B. Müller

**Affiliations:** 1 Translational Psychiatry, Department of Psychiatry and Psychotherapy, Johannes Gutenberg University Medical Center, Mainz, Germany; 2 Leibniz Institute for Resilience Research, Mainz, Germany; 3 Department of Psychiatry and Behavioral Sciences, Stanford University, Stanford, CA USA

**Keywords:** Learning and memory, Psychiatric disorders

## Abstract

Each year, more than half a billion people in the world are affected by stress-related health disorders. Consequently, there is an urgent need for new insights to guide interventions designed to increase stress resilience. Studies of humans and various animals have uncovered the process of stress inoculation, in which exposure to mild stressors enhances subsequent stress resilience. Here, we investigate whether stress inoculation-induced resilience in mice consistently occurs across a multiplicity of different stress contexts (tests). C57BL/6 J adult male mice were randomised either to stress inoculation training (*n* = 36) or to a non-inoculated, but handled control condition (*n* = 36). Thereafter, indications of coping and resilience were assessed during (i) acute social defeat in a context similar to that used for stress inoculation training, and (ii) fear conditioning and learned extinction in a novel context. Stress inoculation effects were also assessed during (iii) tail-suspension and (iv) open-field tests that each represent milder stressors. Stress-inoculated mice showed more active defence behaviour during acute social defeat, higher sociability before and after defeat, and greater indications of learned extinction of conditioned fear compared to non-inoculated control mice. Stress-inoculated mice also responded with diminished tail-suspension immobility and open-field defecation. Results suggest that stress inoculation protects against various stressors that differ in quality and relative intensity. Stress inoculation research in mice may serve as the basis for mechanistic studies of global resilience in humans.

## Introduction

After decades of research on stress-related mental health disorders, success in reducing their frequency remains humble. Each year, more than half a billion people in the world are affected by stress-related mental health disorders^1^. Consequently, there is an urgent need for new insights to guide interventions designed to improve stress coping and increase human resilience^2–4^.

A promising alternative strategy is to shift the research focus from disease-oriented approaches to health-oriented approaches, investigating stress resilience instead of stress susceptibility. This shift is the result of ample evidence that all individuals change during the process of coping with stressors, and not only those who are susceptible^5^. Change in resilient individuals supports the view that resilience is an outcome, resulting from activation of dynamic mechanisms, and not insensitivity or passiveness to stressors. This process of change sometimes manifests as partial inoculation against the effects of future stressors^6,7^. Stress inoculation is defined as the process by which better coping and resilience to future stressful events is developed, following exposure to mildly stressful experiences early in life^8–14^.

A model of stress inoculation in mice has recently been established^15^. The model is a modified version of the well-established chronic social defeat paradigm^16^. The latter builds upon the knowledge that male mice are territorial, and male residents fight male intruders to evict them from their territory. With this model, the main modifications include shorter durations of sensory interaction between resident and intruder mice, and the absence of physical contact, i.e., no fighting takes place because a mesh wall separates resident and intruder. Such modifications train active coping by providing intruders with control, which in turn promotes coping against potential negative effects of future stressors^17^. Interactive sessions take place every second day, allowing sufficient time for rest and memory consolidation^15^. In line with the chronic social defeat model, intruder mice encounter a new resident stranger for each interaction session, maintaining the situation as unpredictable, and no stress habituation takes place^18^. Brockhurst and colleagues found that stress inoculation training acutely increases plasma levels of corticosterone, providing evidence of challenge for the stress-response system. Subsequent behavioural tests, including the tail-suspension test, open-field test, and novel object recognition test, showed enhanced active coping and better stress resilience in inoculated mice compared to non-inoculated controls^15^.

Recently, Kalisch and colleagues proposed different types of stress resilience mechanisms in humans. One mechanism of particular relevance is global resilience. Global resilience mechanisms protect against functional impairments induced by different stressors^2^. Thus, such mechanisms are activated in the face of stressors that differ in their qualities and relative intensities. The behavioural tests conducted by Brockhurst and colleagues were stress contexts that differed from inoculation training sessions, suggesting that induced stress resilience extends to different stress contexts generally considered to be mildly stressful. Accordingly, we investigate here whether induced stress resilience in this model extends to other stress contexts that are more intense. First, acute social defeat was used as a stress context that resembles the social origins of stress inoculation training sessions. Second, we employed fear conditioning and learned extinction as a novel stress context. Additionally, in line with Brockhurst and colleagues, we reexamined inoculation effects in milder novel stress contexts, specifically with tail-suspension and open-field tests.

The study of resilience is not simply a mirror of the study of stress vulnerability; mechanisms of susceptibility and resilience overlap but only partially^19^. Thus, understanding the neurobiology of resilience may provide a more comprehensive picture in the service of understanding psychopathology, treatment development, and prevention. If stress inoculation protects against more than one stress context (strong, mild, social, and physical), this will provide evidence for global resilience mechanisms.

## Materials and methods

**Fig. 1:**
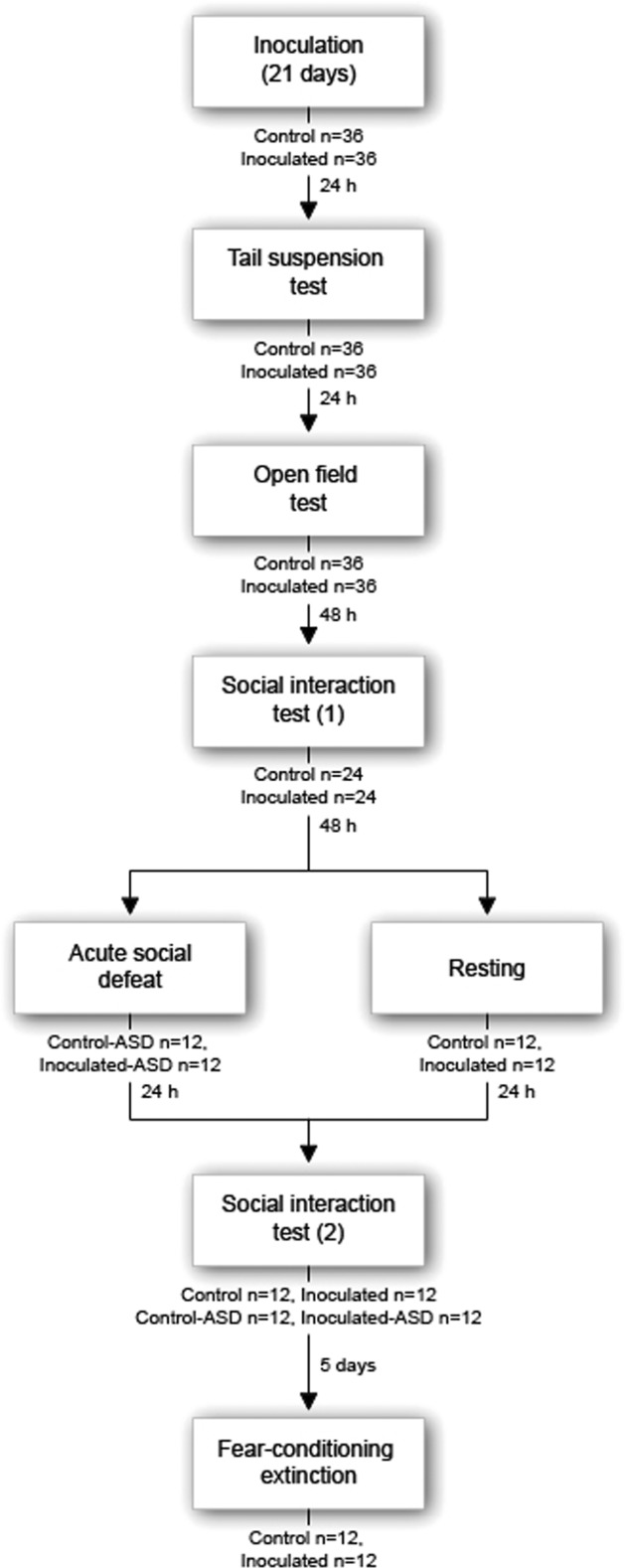
Experiment timeline. Mice underwent inoculation (inoculated) sessions every second day for 21 days, resulting in a total of 11 sessions (*n* = 36 per group). Tail-suspension test followed the termination of the last session by 24 h that in turn was followed by open-field test 24 h later. Following open-field test by 48 h, 24 mice of each group were randomly selected for the remainder of the experiment. The first social interaction test took place followed by acute social defeat 48 h later on *n* = 12 per group (control-ASD and inoculated-ASD). The remaining mice (*n* = 12 per control and inoculated) were left to rest. The second social interaction test followed 24 h later on all mice that in turn was followed 5 days later by fear conditioning/extinction only on the mice that underwent resting earlier, i.e., did not undergo acute social defeat (*n* = 12 per control and inoculated).

Figure 1 represents the experiment’s timeline.

### Animals

C57BL/6 J male mice weighing between (22–28 g) at the age of 7 weeks were obtained from Janvier (France). Mice were housed individually and maintained in a temperature- and humidity-controlled facility on a 12 h light–dark cycle (lights on 8:00; lights off: 20:00; 23 °C; 38% humidity), with food and water ad libitum. All treatments and tests were conducted during the mice’s light phase between 8:30 and 13:30. Sample size was chosen using G-power statistics based on an alpha of 0.05 and a beta of 0.02 (statistical power of 80%). In compliance with animal welfare guidelines, large sample size was chosen over experimental replication. All procedures were performed in accordance with the European Communities Council Directive regarding care and use of mice for experimental procedures, and were approved by local authorities (Landesuntersuchungsamt Rheinland-Pfalz).

### Inoculation training sessions

A total of 72 mice arrived to the facility, after 1 week of habituation, 36 mice underwent inoculation sessions (inoculated) as described elsewhere^15^. In brief, mice were introduced into the cages of larger, older, and retired male breeders (aggressive) from the CD-1 strain (pre-existing in the facility), with a mesh wall between the two all the time for 15 min allowing only sensory contact. Following the sessions mice were returned to their home cages. Sessions took place every second day for 21 days, resulting in a total of 11 sessions with inoculated mice never encountering the same aggressor twice. Age-matched mice maintained in the same conditions but randomised to the non-inoculated control group (*n* = 36) were left undisturbed throughout the 21 days except for regular facility handling (changing cages). Following the termination of the sessions by 24 h mice underwent a behavioural test battery. Light conditions in all tests were 37 lx except for fear conditioning (see below).

### Tail-suspension test

Mice were hung from their tail at a height of 80 cm for 6 min and immobility’s duration was scored. Open-field test followed 24 h later.

### Open-field test

Mice were introduced into open-field arenas (total size 45 cm²) for 5 min and left to explore. The centre area of each arena was considered 10 cm away from the walls. Total distance travelled, time spent at the centre, and faeces count were scored.

Following the open-field test by 48 h, *n* = 24 per group (inoculated and control mice) were randomly selected for the remainder of the experiment.

### First social interaction test

The test was conducted in the same open-field arenas with a mesh enclosure at the centre as described before^16^. In brief, mice were introduced for 2.5 min with an empty mesh enclosure during a habituation phase followed by immediate re-introduction for 2.5 min, with a novel social target under the mesh enclosure during the testing phase. The interaction zone was considered to be 2 cm around the mesh enclosure’s boundaries, and time exploring the novel social target was scored. Acute social defeat followed 48 h later.

### Acute social defeat

Out of the 48 mice that underwent first social interaction test, randomly chosen 12 per group underwent acute social defeat (inoculated-ASD and control-ASD mice), whereas the remaining (*n* = 12 per inoculated and control mice) were left undisturbed (Fig. 1). Mice were introduced into the cages of the aggressors without any mesh wall in between resulting in a fight. The test lasted 5 min and total time of active defence behaviour (fighting back and escaping when attacked) was scored manually by an observer without knowledge of the training treatment conditions. The second social interaction test followed 24 h later and was conducted like the first. Fear conditioning/extinction in turn followed 5 days later.

### Fear conditioning/extinction

Took place in Multiconditioning Box by TSE (Bad Nauheim, Germany) and only with the mice that did not undergo acute social defeat earlier (*n* = 12 per inoculated and control; Fig. 1). Arenas were rectangular (23 × 38 cm) with white walls, metal grid floor, and had lightning conditions of 125 lx.

#### Conditioning training sessions

Conditioning training sessions were a total of four repetitions with 30 s interval between every repetition. The sequence was as follows: 60 s habituation, 60 s light, 30 s of sound physically pulsed into 200 ms with an intensity of 72 db, and frequency of 9000 Hz, during the last 2 s of the sound an electric stimulus (foot shock) of 0.7 mA was given. This sequence was repeated four times before a final delay of 60 s.

#### Testing for context conditioning

Testing for context conditioning followed conditioning sessions by 24 h and consisted of 500 ms habituation followed by light for 180 s, and a final delay of 500 ms.

#### Extinction sessions

Extinction sessions followed testing by 24 h for the next 2 days and involved three repetitions within each day with 30 s intervals between every repetition. The sequence was as follows: 30 s of habituation then three times repetition of 30 s light followed by 30 s sound, finally a delay of 30 s took place. The final repetition of the second day was considered conditioning context recall test of extinction sessions.

### Tracking

Video Tracking and automatic scoring was done using Ethovision software 11.0 by Noldus® (Wageningen, The Netherlands). Automatic scoring was corrected for nose-tail switch errors manually by an observer without knowledge of the training treatment conditions. Nose point was taken for assessing exploration in social interaction tests. Centre point of the body was taken to assess immobility in tail-suspension test and presence in specific zones in open-field test. In the case of fear conditioning, freezing was detected by infrared beams using Multiconditioning Box by TSE (Bad Nauheim, Germany).

### Statistics

All statistical analysis was performed using GraphPad Prism software (version 6). Similar variance between the groups being statistically compared was confirmed before conducting any analysis and all tests performed were two sided. Time was scored in percent of total time of the respective test (percent time).

Tests comparing performance between both groups (inoculated versus control mice) were done using the Mann–Whitney test (tail-suspension test, open-field test, first and second social interaction test, acute social defeat, context-conditioning test of fear conditioning, and its extinction recall). Moreover, comparisons within one group (second social interaction test versus the first, and last phase of extinction sessions versus the first) were done using the Wilcoxon signed-rank test. Freezing time across fear-conditioning sessions was analysed using the Friedman test followed by Dunn’s multiple comparison test. Finally, correlations were assessed using the non-parametric Spearman’s rank correlation coefficient.

## Results

All results presented as mean ± s.e.m. In the graphs’ legends, the number of the samples analysed (*n*), the statistical test of choice, and the *p*-value for each experiment are indicated. Moreover, on the graphs, *p*-values are also indicated as follows: *p* ≤ 0.05*, *p* < 0.01**, and *p* < 0.001***.

### Tail-suspension test

**Fig. 2:**
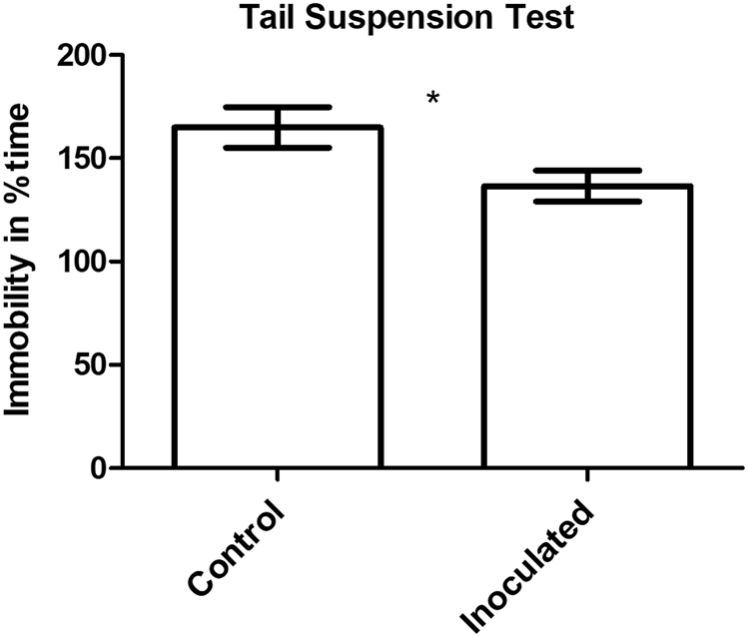
Tail-suspension test. Inoculated mice spent significantly less percent time immobile compared to control mice. Results presented as mean ± s.e.m, *p* ≤ 0.05*, Mann–Whitney test, *n* = 36 per group.

Inoculated mice spent significantly less percent time immobile (i.e., more struggling) as a measurement of active coping compared to control mice (Fig. 2). The same results were found when animals that subsequently underwent ASD or resting were analysed separately (data not shown).

### Open-field test

**Fig. 3:**
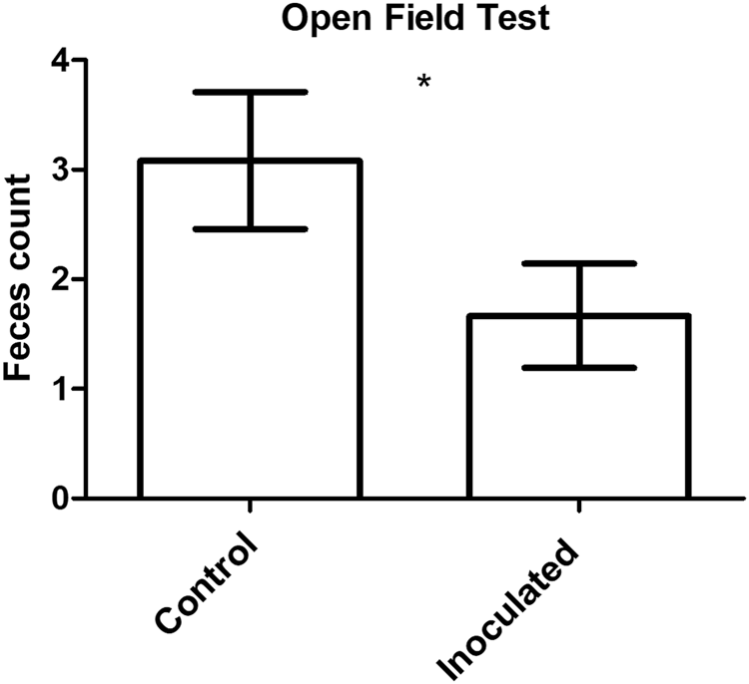
Open-field test. Inoculated mice had significantly lower faeces count compared to control mice. Results presented as mean ± s.e.m., *p* ≤ 0.05*, Mann–Whitney test, *n* = 36 per group.

With respect to basal activity, total distance moved between both groups in the open-field test was similar (data not shown). Percent time spent at the centre area of the open-field arena was scored as a measurement of anxiety, and was similar between both groups (data not shown). However, faeces count as a measurement of emotionality, anxiety, and stress^20–22^, were significantly diminished for inoculated compared to control mice (Fig. 3). When animals that subsequently underwent ASD or resting were analysed separately, significant differences between inoculated and control mice were not found (data not shown). Only when both ASD and resting subsets of animals were analysed together does the sample size become sufficient for reaching statistical significance.

### Social interaction tests and acute social defeat

**Fig. 4:**
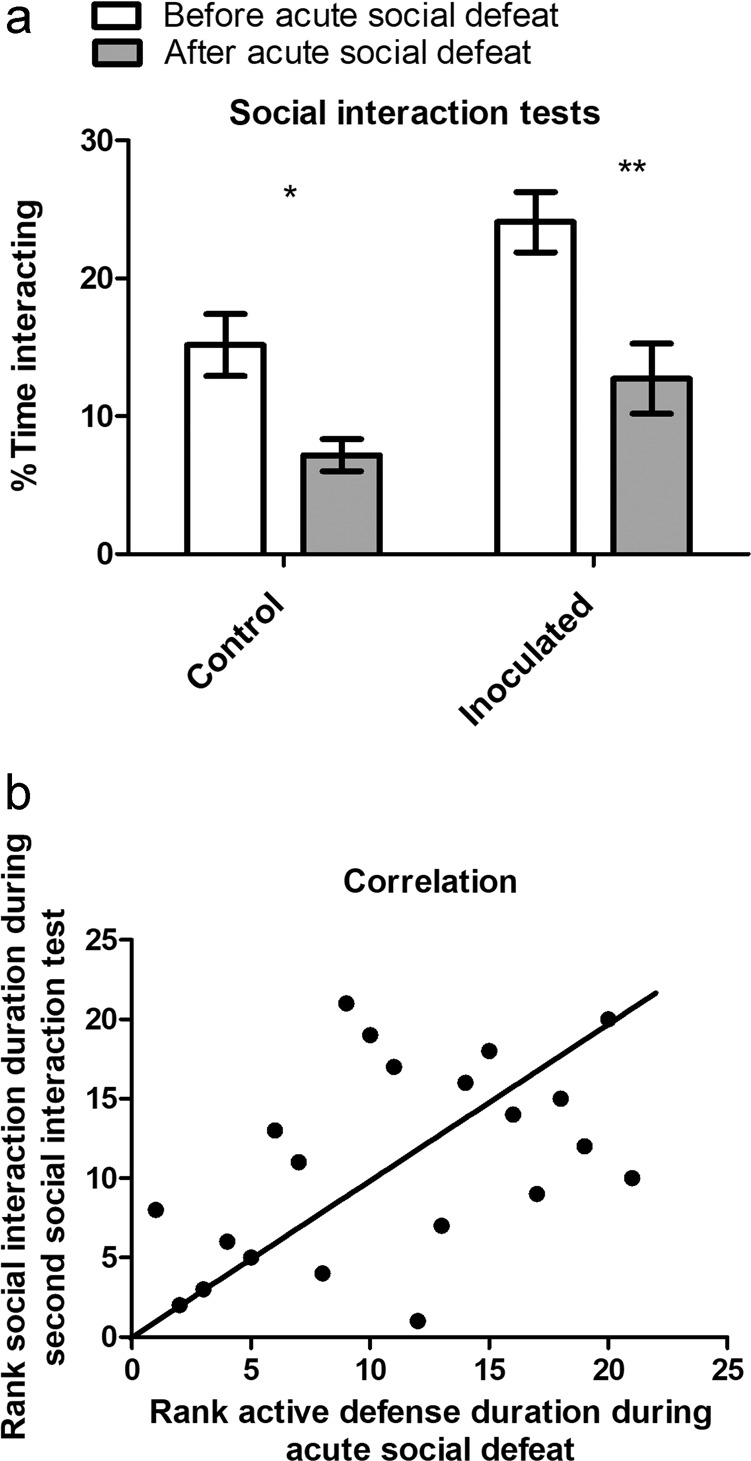
Social interaction tests. **a** Social interaction tests: percent time interacting with the novel social target following acute social defeat was significantly less for both groups compared to before the defeat experience. Moreover, the Mann–Whitney test revealed that: (1) inoculated mice spent significantly greater (*p* ≤ 0.05*) percent time interacting with the novel social target compared to control mice before acute social defeat. (2) On average, percent time interacting with the novel social target was significantly greater (*p* < 0.01**) for inoculated mice compared to control mice. **b** Correlation: percent time defending during acute social defeat significantly positively correlated with percent time interacting with the novel social target presented during the second social interaction test taking place following the defeat. Results presented as mean ± s.e.m, **a**, **b***p* ≤ 0.05*, **a***p* < 0.01**, **a** Wilcoxon signed-rank test within each group, **b** non-parametric Spearman’s rank correlation coefficient, *r* = 0.5, **a***n* = 12 per group, **b***n* = 24.

Inoculated mice spent significantly greater (*p* ≤ 0.05*) percent time interacting with the novel social target compared to control mice during the first social interaction test, i.e., before acute social defeat (Fig. 4a). The greater trend was maintained for inoculated mice during the second social interaction test, i.e., following acute social defeat, but did not reach statistical significance (Fig. 4a). Both groups spent significantly less percent time interacting, following acute social defeat compared to before the defeat experience (Fig. 4a). During both social interaction tests, inoculated mice spent, on average, significantly greater (*p* < 0.01**) percent time interacting compared to control mice (Fig. 4a). Moreover, acute social defeat results indicated that inoculated mice, on average, spent twice the percentage of time defending compared to control mice; however, this difference did not reach statistical significance (data not shown). Finally, percent time defending during acute social defeat significantly and positively correlated with percent time interacting with the novel social target following defeat, i.e., during the second social interaction test (Fig. 4b).

### Fear conditioning/extinction

**Fig. 5:**
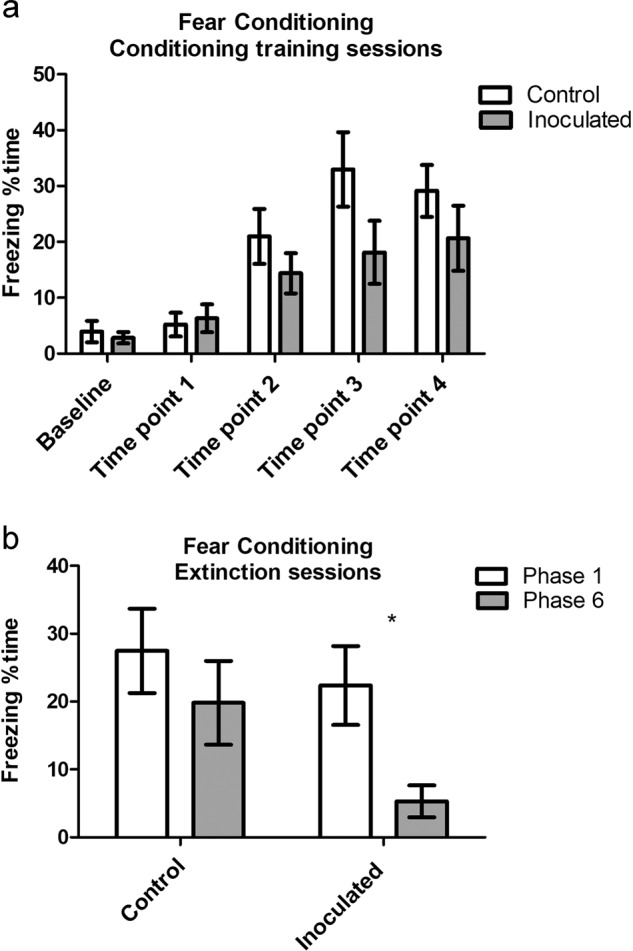
Classical fear conditioning/extinction. **a** Fear conditioning training sessions: percent time freezing significantly increased from baseline to the fourth training time point in the conditioning context (context where foot shocks were received) for inoculated mice and control mice. **b** Extinction sessions: control mice spent similar percent time freezing during the first extinction phase and the last (sixth), whereas inoculated mice spent significantly less percent time freezing in the last phase of extinction compared to the first phase. Moreover, the Mann–Whitney test revealed that inoculated mice spent significantly less (*p* ≤ 0.05*) percent time freezing compared to control mice during only the last extinction phase (sixth). Results presented as mean ± s.e.m, **a***p* < 0.001*** and *p* < 0.01**, **b***p* ≤ 0.05*, **a** Friedman test, **b** Wilcoxon signed-rank test within each group, *n* = 12 per group.

Both groups showed a similar significant increase in percent time freezing as a measurement of learning throughout conditioning training sessions in the conditioning context, where foot shocks were received (Fig. 5a). Results were similar between both groups during testing for context conditioning (data not shown). However, only inoculated mice extinguished the fear memory associated with the conditioning context as measured by a significant reduction in percent time freezing between the first and last (sixth) extinction phases (Fig. 5b). On the other hand, control mice showed no change in percent time freezing between the first and last phases of extinction (Fig. 5b). Moreover, there was a significant difference in percent time freezing between control mice and inoculated mice during the last phase of extinction, which was considered as the conditioning context recall test of extinction, where inoculated mice spent a significantly smaller (*p* ≤ 0.05*) percentage time freezing (Fig. 5b) compared to control mice. Results were similar between both groups during the first phase of extinction.

## Discussion

Stress inoculation-induced resilience occurs consistently across a multiplicity of different stress contexts in mice. Stress-inoculated mice show more active defence behaviour during acute social defeat, higher sociability before and after defeat, and greater indications of learned extinction of conditioned fear compared to non-inoculated control mice. Stress-inoculated mice also exhibit decreased tail-suspension immobility and diminished open-field defecation relative to control mice. Stress inoculation training protects against the deleterious effects of diverse stressors that differ in quality and intensity. Insights gained from stress inoculation research in mice may help to advance translational studies designed to enhance global resilience in humans.

In humans, stress inoculation training is conducted to protect against the deleterious effects of stressors that resemble those that are mimicked or simulated during inoculation training^23^. This aspect of stress inoculation training was investigated in mice with acute social defeat, as a standard stressor that resembles the social features of inoculation training. During acute social defeat, more active defence was observed in stress inoculated compared to control mice, and active defence correlated with sociability during a subsequent encounter with an unfamiliar stranger. Greater sociability in stress-inoculated mice before and after acute social defeat may reflect increased curiosity. Greater curiosity after stress inoculation training is in line with our previous findings^10,15^, and may function as part of a positive feedback process that motivates individuals to seek situations that amplify the effects of stress inoculation training^14,24^. Rodent research further suggests that attempts to escape attack or fight back promote active coping and resilience^25^. Thus, active defence during social defeat fits conceptually with greater sociability insofar as both may reflect resilience-promoting factors^26^.

Another stress context that we investigated after inoculation training was fear conditioning and learned extinction. Unlike social defeat, conditioning and extinction differ significantly from procedures employed during inoculation training. All mice learned the association between a conditioned stimulus and subsequent unconditioned foot shock, and all mice initially exhibited similar conditioned responses after the first extinction session. However, only stress-inoculated mice extinguished conditioned freezing within six extinction sessions. Stress-inoculated mice also struggled more than control mice on tail-suspension tests of active coping^27^, and likewise defecated less during open-field tests as a measurement of diminished emotionality^20–22^. It is possible that extinction of conditioned fear in control mice may have occurred with additional extinction sessions, but extinction of conditioned fear is significantly more rapid after stress inoculation training.

Extinction involves learning of associations between observed events resulting in new conditioned stimulus-unconditioned stimulus expectations^28^. Similar to extinction training, stress inoculation training involves prolonged and repetitive exposure to events, resulting in an experience-dependent learning process. Our findings show that extinction can be enhanced by stress inoculation training and suggest that stress inoculation-induced coping is the result of learning and memory mechanisms that may overlap with those of extinction. At the molecular level, one such mechanism is mitogen-activated protein kinase (MAPK)/extracellular signal-regulated kinase (ERK) signalling. ERK signalling in basolateral amygdala, dorsal hippocampus, and medial prefrontal cortex mediates acquisition of extinction memory^29^. Moreover, functionally different compounds that produce rapid and long-lasting antidepressant effects, are reported to stimulate ERK signalling in the brain^30^. In fact, a recent study reported that the induced activation of ERK by one such antidepressant, specifically ketamine, enhanced the expression of extinction memory in mice^31^. Similar findings were reported following the administration of a selective delta opioid receptor agonist (KNT-127), where MAPK/ERK signalling in the amygdala and the hippocampus was a key mediator of enhanced extinction learning^29^. Both, antidepressants and delta opioid receptors play an important role in the regulation of emotions^32^, rendering them attractive candidates for investigating underlying molecular mechanisms of stress inoculation-induced enhancement of extinction.

Certain molecular targets modulated by learning and memory also mediate early life effects on cognitive reserve, which has been linked to flexibility in brain functions^33^. The cognitive reserve hypothesis suggests that early life experiences programme the brain by inducing persistent morphological changes in regions critical for learning and memory^34–38^, and for altered hypothalamic–pituitary–adrenal (HPA) axis reactivity resulting in long-lasting effects on sensitivity to future stressors^39^. Stress inoculation-induced coping may result from similar cognitive reserve effects induced by prior training experiences. Specifically, our finding of enhanced learned extinction after stress inoculation training reflects increased cognitive flexibility, and reduced HPA axis reactivity to restraint stress after the same stress inoculation training protocol employed here reflects a new balance of HPA axis reactivity^15^. Taken together, these findings suggest that stress inoculation training may enhance cognitive reserve, which in turn improves coping across multiple stress contexts.

Extinction of conditioned fear is particularly attractive for stress resilience research because this process is impaired in patients with anxiety disorders, and aspects of learned extinction resemble exposure therapy for anxiety^40,41^. Whereas exposure therapy is employed as a treatment for anxiety, stress inoculation training is often considered a preventive intervention. Chronic stress is known to impair extinction of conditioned fear^42^, and the absence of such impairments indicates that stress inoculation training differs from exposure to chronic stress.

A limitation of our research includes the size of samples used to study acute social defeat. With larger samples, we expect that differences in active defence will reach statistical significance because greater variability in this measurement was evident in stress inoculated versus control mice. Another limitation is that only males were investigated. Stress-related mental health disorders are prevalent in men and women, but certain disorders, such as, depression are more prevalent in women^43^. Although indications of resilience induced by stress inoculation have been recently reported for female mice^44^, further studies are needed to extend the findings reported here from male to female mice. Moreover, the findings reported here are from young adult mice. Stress inoculation training during early life has been reported in squirrel monkeys^10,14,24^. Compared to primate models, mouse models allow more tools to dissect causal mechanisms mediating experience-dependent learning. It would add to our understanding to know whether the findings of induced stress-coping effects are restricted to young adults or extend to different developmental stages in mice, i.e., is there an optimal time window for stress inoculation treatment. Finally, global resilience, as proposed by Kalisch and colleagues, refers to protection from different stressors and protection against various dysfunctions. Here, we focus on resilience in the face of different stressors because disorders or dysfunctions in human mental health may or may not have valid counterparts in mice.

In summary, we show that stress inoculation training protects mice against deleterious effects of diverse stressors that differ in quality and relative intensity. Studies of mice may help to advance translational research designed to enhance global resilience in humans. The possibility that overlapping mechanisms of conditioned learning and memory mediate stress inoculation and learned extinction is an interesting question for future research. A first step would be to investigate whether the relation between stress inoculation training sessions and more rapid extinction of conditioned fear is described by a learning curve. Learning curve data will reveal whether stress inoculation effects are mediated by learning, and will also provide information about the speed at which beneficial effects are achieved and insights on how to improve them. Learned extinction research is remarkably coherent^45^ and may offer mechanistic insights on stress inoculation-induced indications of global resilience.

## References

[1] Vos T (2015). Global, regional, and national incidence, prevalence, and years lived with disability for 301 acute and chronic diseases and injuries in 188 countries, 1990–2013: a systematic analysis for the Global Burden of Disease Study 2013. Lancet.

[2] Kalisch R, Müller MB, Tüscher O (2015). A conceptual framework for the neurobiological study of resilience. Behav. Brain Sci..

[3] Horn SR, Charney DS, Feder A (2016). Understanding resilience: new approaches for preventing and treating PTSD. Exp. Neurol..

[4] Nijhof SL (2018). Healthy play, better coping: the importance of play for the development of children in health and disease. Neurosci. Biobehav. Rev..

[5] Russo SJ, Murrough JW, Han M-H, Charney DS, Nestler EJ (2012). Neurobiology of resilience. Nat. Neurosci..

[6] Seery MD, Holman EA, Silver RC (2010). Whatever does not kill us: cumulative lifetime adversity, vulnerability, and resilience. J. Pers. Soc. Psychol..

[7] Seery MD, Leo RJ, Lupien SP, Kondrak CL, Almonte JL (2013). An upside to adversity? Moderate cumulative lifetime adversity is associated with resilient responses in the face of controlled stressors. Psychological Sci..

[8] Dienstbier RA (1989). Arousal and physiological toughness: implications for mental and physical health. Psychological Rev..

[9] Khoshaba DM, Maddi SR (1999). Early experiences in hardiness development. Consulting Psychol. J. Pract. Res..

[10] Parker KJ, Buckmaster CL, Schatzberg AF, Lyons DM (2004). Prospective investigation of stress inoculation in young monkeys. Arch. Gen. Psychiatry.

[11] Fox C, Merali Z, Harrison C (2006). Therapeutic and protective effect of environmental enrichment against psychogenic and neurogenic stress. Behavioural Brain Res..

[12] Rutter M (2006). Implications of resilience concepts for scientific understanding. Ann. N. Y. Acad. Sci..

[13] Meichenbaum, D., Carlson, J., & Kjos, D. *Cognitive-Behavioural Therapy* (American Psychological Association, 2007).

[14] Lyons DM, Parker KJ, Katz M, Schatzberg AF (2009). Developmental cascades linking stress inoculation, arousal regulation, and resilience. Front. Behav. Neurosci..

[15] Brockhurst J, Cheleuitte-Nieves C, Buckmaster CL, Schatzberg AF, Lyons DM (2015). Stress inoculation modeled in mice. Transl. Psychiatry.

[16] Golden SA, Covington HE, Berton O, Russo SJ (2011). A standardized protocol for repeated social defeat stress in mice. Nat. Protoc..

[17] Benight, C. C., & Cieslak, R. In *Resilience and Mental Health: Challenges Across the Lifespan*, Ch. 3 (Cambridge University Press, 2011).

[18] Herman JP (2013). Neural control of chronic stress adaptation. Front. Behav. Neurosci..

[19] Liberzon I, Knox D (2012). Expanding our understanding of neurobiological mechanisms of resilience by using animal models. Neuropsychopharmacology.

[20] Crofton EJ, Zhang Y, Green TA (2015). Inoculation stress hypothesis of environmental enrichment. Neurosci. Biobehav. Rev..

[21] Flint J (1995). A simple genetic basis for a complex psychological trait in laboratory mice. Science.

[22] Gould, T. D., Dao, D. T., & Kovacsics, C. E. The Open Field Test. In *Mood and Anxiety Related Phenotypes in Mice* (ed Gould, T.D.) 1–20 (Humana Press, Totowa, NJ, 2009). https://link.springer.com/protocol/10.1007/978-1-60761-303-9_1.

[23] Cecil MA, Forman SG (1990). Effects of stress inoculation training and coworker support groups on teachers’ stress. J. Sch. Psychol..

[24] Lyons DM, Parker KJ (2007). Stress inoculation-induced indications of resilience in monkeys. J. Trauma. Stress.

[25] Feder A, Nestler EJ, Charney DS (2009). Psychobiology and molecular genetics of resilience. Nat. Rev. Neurosci..

[26] Amstadter AB, Moscati A, Maes HH, Myers JM, Kendler KS (2016). Personality, cognitive/psychological traits and psychiatric resilience: a multivariate twin study. Pers. Individ. Dif..

[27] De Kloet, E. R., & Molendijk, M. L. Coping with the forced swim stressor: towards understanding an adaptive mechanism. *Neural Plast.* 1–13 (2016). 10.1155/2016/6503162 PMC4806646 27034848

[28] Hofmann SG (2008). Cognitive processes during fear acquisition and extinction in animals and humans: implications for exposure therapy of anxiety disorders. Clin. Psychol. Rev..

[29] Yamada D (2019). Selective agonists of the δ-opioid receptor, KNT-127 and SNC80, act differentially on extinction learning of contextual fear memory in mice. Neuropharmacology.

[30] Lepack AE, Bang E, Lee B, Dwyer JM, Duman RS (2016). Fast-acting antidepressants rapidly stimulate ERK signaling and BDNF release in primary neuronal cultures. Neuropharmacology.

[31] Girgenti MJ, Ghosal S, LoPresto D, Taylor JR, Duman RS (2017). Ketamine accelerates fear extinction via mTORC1 signaling. Neurobiol. Dis..

[32] Saitoh, A., & Nagase, H. In *Delta Opioid Receptor Pharmacology and Therapeutic Applications*, 3–19 (Springer, Cham, 2016).

[33] Lesuis. SL (2018). Vulnerability and resilience to Alzheimer’s disease: early life conditions modulate neuropathology and determine cognitive reserve. Alzheimer’s Res. Ther..

[34] Bock J, Gruss M, Becker S, Braun K (2005). Experience-induced changes of dendritic spine densities in the prefrontal and sensory cortex: correlation with developmental time windows. Cereb. Cortex.

[35] Bock J, Murmu MS, Biala Y, Weinstock M, Braun K (2011). Prenatal stress and neonatal handling induce sex-specific changes in dendritic complexity and dendritic spine density in hippocampal subregions of prepubertal rats. Neuroscience.

[36] Murmu MS (2006). Changes of spine density and dendritic complexity in the prefrontal cortex in offspring of mothers exposed to stress during pregnancy. Eur. J. Neurosci..

[37] Kolb B (2012). Experience and the developing prefrontal cortex. Proc. Natl Acad. Sci. USA.

[38] McEwen BS, Eiland L, Hunter RG, Miller MM (2012). Stress and anxiety: structural plasticity and epigenetic regulation as a consequence of stress. Neuropharmacology.

[39] Vallee M (1997). Prenatal stress induces high anxiety and postnatal handling induces low anxiety in adult offspring: correlation with stress-induced corticosterone secretion. J. Neurosci..

[40] Milad MR (2008). Presence and acquired origin of reduced recall for fear extinction in PTSD: results of a twin study. J. Psychiatr. Res..

[41] Jovanovic T, Norrholm SD (2011). Neural mechanisms of impaired fear inhibition in posttraumatic stress disorder. Front. Behav. Neurosci..

[42] Hoffman AN, Lorson NG, Sanabria F, Foster Olive M, Conrad CD (2014). Chronic stress disrupts fear extinction and enhances amygdala and hippocampal Fos expression in an animal model of post-traumatic stress disorder. Neurobiol. Learn. Mem..

[43] World Health Organization. *Gender and Mental Health.* https://apps.who.int/iris/bitstream/handle/10665/68884/a85573.pdf?sequence=1&isAllowed=y (2002).

[44] Lyons DM, Buckmaster CL, Schatzberg AF (2018). Learning to actively cope with stress in female mice. Psychoneuroendocrinology.

[45] Milad MR, Quirk GJ (2012). Fear extinction as a model for translational neuroscience: ten years of progress. Annu. Rev. Psychol..

